# Report of the Munich Veterinary Institute for the Year 1883

**Published:** 1884-10

**Authors:** 


We have before us the "Seventh Supplement" to the "Deutsche Zeitschrift für Thiermedicin," which is the Annual Report of the Munich Veterinary Institute, for the year 1883. This Report covers 182 pages of very interesting matter, and is of especial value showing what can be done by a small, well

*From the Deutsche Zeitschrift für Thiermedicin, Vol. 10, Part I.

regulated school endowed by the State in comparison with the kind of institutions we have in this country.

The Faculty of the School consisted of:

1. Director, Dr. Ludwig Franck (since deceased), teacher and demonstrator of anatomy, veterinary police and horse-shoeing.
2. Prof. Karl Hahn, teacher of surgery and conductor of the surgical clinic.
3. Johan Feser, professor of chemistry, physic, toxicology, materia medica, etc.
4. Dr. Friedberger, professor of special pathology and therapeutics, exterior, and conductor of the inner clinic.
5. Dr. Tappenheimer, professor of physiology and dietetics.
6. Dr. Harz, professor of botany and zoölogy.
7. Dr. Bonnet, professor of histology, embryology, general pathology and pathological anatomy.

ASSISTANTS.

8. Dr. Gutenäcker, teacher of practical horse-shoeing.
9. Dr. Kitt, assistant in anatomy.
10. Dr. Fröhner, clinical assistant.
11. Dr. Kleber, chemical and pharmaceutical assistant.
12. Dr. Stoss, assistant in pathological anotomy.
13. Dr. Beichhold, second clinical assistant.
14. Dr. Martin, second assistant in pathological anatomy.
15. Dr. Robl, director of the abattoir, and lecturer on meat and market inspection.

The following scientific and literary contributions have emanated from the teachers of this school during the year 1883, which compares very favorably with the nihilistic silence with which the schools of English speaking countries have deported themselves during the same period. The fault is not with our schools, however, but with the system upon which they are founded. Such work can *never* be attained except with government support and the small pay and pension system.

From Prof. Franck, we have a second and revised edition of his valuable text-book on Zoötomy—the best in the German language. Also, a compendium of the same.

From Prof. Feser, a popular discussion of Breeding in Cattle; as well as a Report of the Inspection of Animals for Contagio-infectious Diseases in the district of Regensburg for 1883.

From Friedberger, a Report of the Internal Clinic during 1883.

From Dr. Horz, several experimental Reports upon Clover and Grasses as articles of Animal Food.

From Bonnet, Report of the Work done in the Pathological and Embryological Laboratory; also, 1, A Contribution to the Embryology of the Ruminants, especially Sheep. 2. The Diseases of the Animals held for sporting purposes, Deer, Bear, Rabbit, etc. 3. The Study of Development in the Egg of the Bird. 4. Studies of Development in the Ovum of Sheep. 5. Ichthyological Contributions.

From Dr. Kitt. 1. Study of the Papillae of the Milk-glands in the Domestic Animals. 2. A Contribution to the Anatomy and Physiology of the Lachrymal Ducts in Horses and Cattle. 3. Contribution to the Study of the Brain in the Domestic Animals. 4. Pachymeningitis spinalis in the Dog—assisted by Dr. Stross. 5. Amyloid Infiltration in the Tuberculosis of Fowls. 6. Odontological Notices. 7. Horse and Tapir; a prehistoric sketch. 8. Histological Researches in Foot and Mouth Disease. 9. A New Nematode in Swine.

Here let me remark, that these contributions of my friend, Dr. Kitt, show what is possible for a young man to do under the Continental School System. Such work as this is impossible in the United States unless a graduate has personal means sufficient to support him in his work. Scientific ability and enthusiasm avail an American nothing. We have them both in quite a number of men in each year's class, but the opportunity and means to work both fail them utterly.

From Dr. Martin. 1. A Contribution to the Study of nodular and calcareous Formations in the Lungs of the Horse. 2. A Contribution to the Development of the Feel-Hairs in the Domestic Animals.

During the year, 503 cadavers were submitted to microscopic examination, and 300 new specimens added to the Pathological Museum. Various new instruments were added to the different laboratories, and the Chirurgical Armatum supplied with all the necessary improvements. The number of students was 42 regular and 13 Hospitants, that is, Agriculturists, etc., who take special lectures.

In his report of the workings of the Inner Clinic, Friedberger gives us valuable papers upon Infectious Fibrinous Pneumonia in the Horse; Stomatitis Pustulosa Contagiosa in the Horse; Colic in the Horse; Oxyuris Mastigodes in the Horse; and a malignant form of Catarrhal Fever in Sheep.

Dr. Martin describes numerous pathological specimens and changes met with in the organs of animals in his pathological report.

Franck gives us a very valuable contribution upon Inoculation for the Prevention of Anthrax; a translation of which will appear in our next number.

